# Tobacco cessation mobile app intervention (Just Kwit! study): protocol for a pilot randomized controlled pragmatic trial

**DOI:** 10.1186/s13063-019-3246-2

**Published:** 2019-02-26

**Authors:** Kar-Hai Chu, César G. Escobar-Viera, Sara J. Matheny, Esa M. Davis, Brian A. Primack

**Affiliations:** University of Pittsburgh, 230 McKee Place, Suite 600, Pittsburgh, PA 15213 USA

**Keywords:** Young adult, Smoking cessation, Tobacco, Smartphone

## Abstract

**Background:**

Adolescence and young adulthood are critical times of initiation and progression to daily use of tobacco. However, it is difficult to recruit young adults to traditional smoking cessation and retention rates are typically low. Smartphone cessation applications (apps) can provide real-time responses to smoking urges and related cues, which are known to be important factors in lapse and relapse. Given the popularity of smartphones among young adults and the considerably higher download rates of commercially developed apps compared to research-based apps, there is a need to design pragmatic studies that evaluate commercial tobacco cessation apps. The aims of this pilot study are to assess the impact on tobacco cessation of using a smartphone app compared with usual care and to generate feasibility data to inform a future fully powered clinical trial.

**Methods:**

We will conduct an open randomized controlled trial with parallel groups. Participants will be selected from hospitalized patients and must be aged 18–30 years, interested in cessation, smoked > 5 cigarettes/day over the past 30 days, and own an Apple or Android smartphone. Participants who are eligible will be randomized to either a smartphone experimental group or patient-initiated follow up (usual care). As this study seeks to assess feasibility, the primary data will include (1) recruitment rates, (2) retention rates, and (3) adherence, measured through user engagement with the app.

**Discussion:**

This pilot trial will be the first to evaluate a commercially available smartphone app for tobacco cessation in a hospitalized setting. Data generated by this study can be used for larger fully powered trials such as comparative effectiveness studies against apps developed by academics or health scientists based on behavioral theories, or cost-effectiveness analyses of mobile interventions.

**Trial registration:**

ClinicalTrials.gov, NCT03538678. Registered on 28 May 2018.

**Electronic supplementary material:**

The online version of this article (10.1186/s13063-019-3246-2) contains supplementary material, which is available to authorized users.

## Background

Tobacco use continues to be the leading cause of preventable death in the USA [1]. Smoking causes almost 9 out of 10 lung cancers. Indeed, the US Surgeon General’s Report notes that young adults’ smoking remains a major public health concern [2]. However, individuals who successfully quit smoking reduce their risk of death from lung cancer by half within 10 years. Although 30 million smokers contact a healthcare provider each year [3], and more than half attempt to quit, most are not successful [4]. Additionally, most available treatments are not optimized for use among the young adult population [5], and unfortunately, young adults who try to quit often relapse [6]. Adolescence and young adulthood remain critical times of initiation and progression to daily use of tobacco [7]. Nevertheless, there are barriers to effective implementation of traditional smoking cessation programs among young adults. First, it is difficult to recruit young adults into traditional programs [8], and retention rates for this population in these programs are as low as 33% [9]. Moreover, traditional program interactions between facilitators and clients are highly intermittent. This compromises the ability to provide real-time responses to smoking urges and related cues, which are known to be important factors in lapse and relapse.

Smartphones have become ubiquitous in the USA, especially among young adults. The Pew Research Center reported in 2018 that 94% of US adults age 18–29 years owned a smartphone [10]. Mobile health (mHealth) researchers have taken advantage of these opportunities: Android and Apple platforms have numerous options of commercially developed apps designed to help quit tobacco use. However, studies reviewing tobacco cessation apps have been generally descriptive [11–13], providing information such as popularity, features, or techniques used for smoking cessation. In addition, all of these reviews cite a lack of evidence-based research, and no published studies report verified information on any health outcome.

In spite of the lack of evidence, commercially developed apps for tobacco cessation are successful. In comparison, there are two apps available through smokefree.gov, in collaboration with the National Cancer Institute (NCI): QuitGuide and quitSTART. While these apps were developed through research efforts by NCI, they are not highly rated by user reviews and are less popular than commercial apps. Both QuitGuide and quitSTART have each been downloaded approximately 1/10th as many times as Kwit, a leading commercial app and the focus of the current study, according to Google Play. This poor performance of research-based apps may be due to the lack of dedicated marketing teams and/or insufficient updates, as research-based apps can depend on continued funding for support rather than profits. For example, as of this writing, neither app has been updated in several months, while top-rated commercial apps are updated every 1–2 weeks.

Given the popularity of smartphones among young adults and the considerably higher download rates of commercially developed apps compared to research-based apps, there is a need to design pragmatic studies that evaluate commercial tobacco cessation apps. This study aims to fill this gap by combining the advantages of traditional industry-developed software with the benefits of a comprehensive, research-focused feasibility trial.

Moreover, while human-computer systems, including smartphone apps, are usually built to focus on principles of interface design and user engagement [14, 15], this study also offers the opportunity to systematically study the use of an app to understand why it might be effective based on known behavior change theories, techniques, or mechanisms.

### Study objectives

The primary aim of this pilot study is to assess the impact on tobacco cessation of using a smartphone app compared with usual care. The secondary aim is to generate feasibility data to provide the necessary information to inform a future fully powered clinical trial.

## Methods

### Trial design

**Fig. 1:**
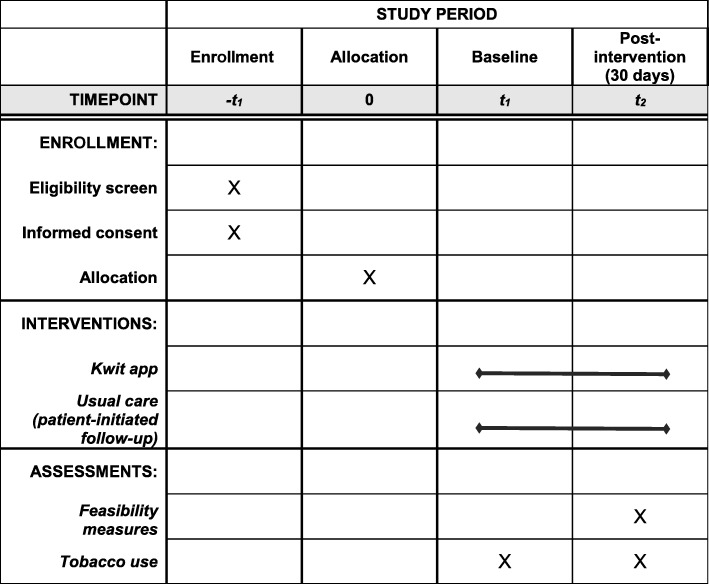
Standard Protocol Items: Recommendations for Interventional Trials (SPIRIT) schedule of enrollment, interventions, and assessments

We will conduct an open randomized controlled trial (RCT) with parallel groups. For additional information on this study’s compliance with Standard Protocol Items: Recommendations for Interventional Trials (SPIRIT), see Fig. 1 and Additional file 1.

### Participants

Participants will be recruited from hospitalized patients from UPMC Montefiore, a large, general hospital located in the mid-Atlantic region of the USA. When patients are admitted, the clinical staff document patient smoking status. Members of the hospital’s Tobacco Treatment Service (TTS) receive a daily list of newly admitted patients who are smokers and attempt to provide approximately 20 min of bedside counseling. A study research staff member will ask permission to shadow the TTS counselor during the session. At the end of the counseling session, if a patient is motivated to quit smoking and meets the inclusion criteria, the research staff member will describe the study, assess eligibility, obtain informed consent (see Additional file 2), collect baseline data, and assign the patient to a study arm. The inclusion criteria are: young adult smoker, aged 18–30 years, interested in cessation, smoked > 5 cigarettes/day over the past 30 days, and must own an Apple or Android smartphone. Potential participants will be excluded if they are already receiving pharmacological and/or behavioral intervention or counseling for tobacco cessation, are unable to provide informed consent, or do not speak English. Participants will be compensated US$25 for completion of each survey for a total of US$50.

### Recruitment

Over a 6-month period, we will recruit 40 young adult smokers aged 18–30 years who are interested in smoking cessation. Participants will receive a TTS counseling session focused on motivational interviewing and then be randomized 1:1 to either standard patient-initiated follow up (through the state Quitline) or use of the Kwit app for 1 month. Participants will be stratified by sex at a 1:1 ratio (10 male and 10 female participants in each group) because men and women are known to have highly variable responses to existing tobacco control interventions [16]. The recruitment procedure will follow existing processes currently being implemented by the TTS. In FY2016, there were 3078 everyday smokers that consented to receive TTS counseling and additional follow up. Of that population, 363 (11.8%) were aged 18–30 years. Therefore, a 6-month recruiting period would expect to identify approximately 181 potential young adult smokers to meet the eligibility criteria. Stratification by sex as planned is highly feasible because roughly half of all TTS-identified individuals were male. While the recruitment of 40 individuals from about 181 who are eligible (22%) seems highly feasible to the TTS team, we have developed contingency plans for recruitment should numbers fall short. Individuals will be compensated for their time to participate in follow-up interviews at the end of 1 month.

### Sample size

Given this is a feasibility trial, a formal sample size calculation is not appropriate [17]. For the current study, we are informed by quantitative usability-testing recommendations that suggest a sample size of 20 [19]. Because we wish to stratify by birth sex, we obtained our sample size of 40 individuals. While we expect this to be more than sufficient, we will remain flexible in our approach and will recruit additional individuals if necessary.

### Randomization

Participants who are eligible will be randomized to either a smartphone experimental group or patient-initiated follow up (usual care) by the research team. They will be stratified by gender, and will be randomized in total using a 1:1 ratio. Neither the participant nor the research team will be blind to their status. An independent statistician will generate an allocation table using Stata’s *ralloc* function. That list will be provided to a member of the research recruitment team, who will upload the table into the Research Electronic Data Capture (REDCap) online survey. Neither the principal investigator (PI) nor any researcher conducting analyses will have access to the randomization process or allocation table. Once recruitment has started, participants that have consented to the study will then be assigned by REDCap to either the experimental or control arms based on the allocation order.

### Intervention

**Fig. 2:**
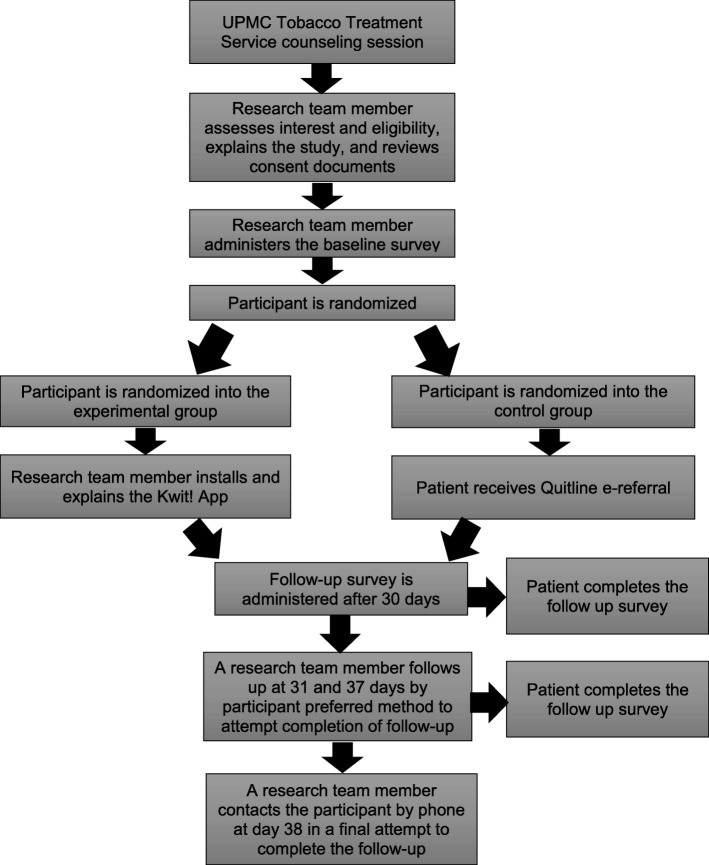
Patient participation flowchart for the Just Kwit study

The Just Kwit! study has two arms (see Fig. 2). For the intervention arm, the Kwit app will be installed on the participant’s phone by a research team member. The team member will help the participant create an account and explain the functions of the Kwit app. App use will be tracked for the 30 days between the baseline and follow-up surveys. The control arm is the current standard of care, which is a TTS consult with patient-initiated follow ups after discharge.

In choosing a smartphone app for this study, the selection criteria were (1) available for both Apple and Android; (2) well-rated in both platforms (i.e., 4+ stars); (3) at least 100,000 downloads; (4) features that are comparable to other available apps; and (5) the development team would be willing to have an open collaboration both in data sharing and software development. The Kwit app matched each criterion; it is one of the few cessation apps that is available for download for both Apple and Android. Kwit is highly rated in both online stores, scoring 4.5/5 for Apple and 4/5 for Android. It has been downloaded 100,000–500,000 times on Android phones (Android releases only ranges for downloads). Kwit combines several popular features of tobacco cessation apps, including game-like features (e.g., unlocking achievement milestones for completing cessation-related tasks), useful statistics (e.g., money saved), and an ability to share progress with friends. Users can actively engage with the app to track cravings or view progress, such as life expectancy gained. A passive component notifies users when milestones have been achieved (e.g., when blood no longer contains nicotine). Motivational messages can be actively requested at any time; they can also appear unsolicited, such as when the user logs cravings. The basic app is free, although users are able to purchase additional features, such as removing ads, if so desired.

As with other commercially developed tobacco cessation apps, Kwit development was not informed by evidence-based practices: its features were based on personal experience and user feedback. However, the two aims of this study will provide data to identify which components are most promising, and if health behavior-change mechanisms can help explain their effect. Connecting to a model of behavior change is important as we seek to modify behaviors among young adults. For example, the motivational messages might be closely tied to motivational enhancement therapy, while the money or lifespan trackers might be related to self-monitoring.

### Outcomes

As this study seeks to assess feasibility, the primary data will include (1) recruitment rates, (2) retention rates, and (3) adherence, measured through user engagement with the app. High usability measures correlate strongly with effective interventions [20]. Engagement with the app will be measured using both survey data in conjunction with logged data from the app itself. Logged data will include specific features that are used as well as patterns of activity (e.g., checking the current money saved after viewing a motivational message). We are particularly interested in the use patterns of two popular features in many cessation apps: calculator and calendar. Measures of usability are based on principles from ubiquitous computing guidelines [21]. These concepts will include (1) attention (e.g., “How many times did you use Kwit per day?”); (2) trust (e.g., “Were you able to complete tasks with ease?”); (3) conceptual model (e.g., “Did Kwit behave as expected?”); (4) interaction (e.g., “Was Kwit frustrating to use?”); and (5) impact (e.g., “How comfortable were you using Kwit in social settings?”). Following guidelines for evaluating the usability of medical applications [22], responses will be either yes/no, or chosen from a Likert scale of 1–9 (a longer Likert-type scale was selected to minimize the risk of a ceiling effect). These questions will be paired with open-ended qualitative questions and comments that will complement numerical ratings. For example, an interaction question will first ask the user: “Was Kwit frustrating to use?” with a 1–9 Likert scale option, followed by the open-ended question: “Please describe an example of how Kwit was frustrating to use.”

### Data collection and management

Data will be collected by a member of the study team both on paper and using the REDCap tools hosted at the University of Pittsburgh [23]. REDCap is a secure, web-based application designed to support data capture for research studies, providing (1) an intuitive interface for validated data entry; (2) audit trails for tracking data manipulation and export procedures; (3) automated export procedures for seamless data downloads to common statistical packages; and (4) procedures for importing data from external sources. Consent documents, payment information, and eligibility screening documents will be in paper form. The two surveys administered in this study, the baseline and the follow up, will be completed within REDCap. The baseline survey will be administered to the patient bedside by a member of the research team directly following enrollment and consent. The follow-up survey will be provided to the participant via email, phone, or in person, depending on the participant’s preference. Only members of the research team will have access to the collected data.

The PI has the primary responsibility for study monitoring. Given the nature of the trial (i.e., brief, limited feasibility), there being no access to protected health information, and there being a limited amount of personal information being collected, no formal data monitoring committee will be convened. Quarterly assessments will be made of data quality and timeliness, participant recruitment, accrual and retention, participant risk/benefit ratio, protection of confidentiality of information, and any other factors that affect the study. The Institutional Review Board (IRB) will be informed immediately on a case by case basis of any adverse outcomes, while requests for modifications of the protocol will be submitted to the IRB on a quarterly basis. Confidentiality of the data and the results of monitoring will be protected.

### Data analysis

The primary method of analysis will be provision of descriptive statistics characterizing key app features, such as viewing of money saved, viewing of quit days, achievement sharing, and crave logging. We will examine descriptive and qualitative data in the context of the fit between individuals, task and technology (FITT) framework [24]. The FITT framework is prevalent among human-computer interaction studies and often used in mobile health research [25]. It posits a strong relationship between individual, task, and technology, and was adopted to assess feasibility and usability in a holistic manner. The FITT approach will help to assess how usability and feasibility metrics are interrelated, and the cyclic effects with relation to the user. We will identify associations between survey Likert-scale responses (i.e., usability and feasibility measures) and quantitatively measured actions from app log data. Given the ordinal data and unknown distributions, the Mann-Whitney U test will be used for statistical analyses.

### Power calculations

In a feasibility pilot trial, it is inappropriate to consider mean outcomes due to the lack of power [17, 18]. However, we will still calculate standard deviation of the differences between the intervention and control groups at 1 month, which is a standard time for tobacco cessation treatment. These data can help inform the calculations needed for a larger RCT.

## Discussion

This pilot trial will be the first to evaluate a commercially available smartphone app for tobacco cessation in a hospitalized setting. The study offers a research/industry collaboration that allows the study to leverage resources from two directions: (1) providing cutting-edge research tools and methods founded on evidence-based academic research and (2) applying translational and implementation strategies from the software development team.

We have chosen a pragmatic approach in order to simulate more real-world settings in app usage. Indeed, the effectiveness of off-the-shelf tobacco cessation apps is compromised by the rapid development cycle of mobile apps and urgency for apps to be quickly released and profitable. Data generated by this study can be used for larger fully powered trials such as comparative effectiveness studies against apps developed by health scientists based on behavioral theories or cost-effectiveness analyses of mobile interventions.

## Trial status

Protocol version: 4

Trial registration: ClinicalTrials.gov

Registration number: NCT03538678 (https://clinicaltrials.gov/ct2/show/NCT03538678)

Date of trial registration: 28 May 2018

Was this trial prospectively registered? Yes

Date recruitment began: June 2018. Recruiting is ongoing

Anticipated completion date: May 2019.

## Additional files

Additional file 1: SPIRIT 2013 checklist. (DOCX 58 kb)

Additional file 2: Informed consent materials. (DOCX 105 kb)

## Abbreviations

FITT: Fit between individuals, task and technology
IRB: Institutional review board
mHealth: Mobile health
NCI: National Cancer Institute
PI: Principal investigator
RCT: Randomized control trial
REDCap: Research Electronic Data Capture
TTS: Tobacco Treatment Service
