# Supplementary material for: Tobacco cessation mobile app intervention (Just Kwit! study): protocol for a pilot randomized controlled pragmatic trial
Source: Trials. 2019 Feb 26;20:147. doi: 10.1186/s13063-019-3246-2 (PMC6390617; doi:10.1186/s13063-019-3246-2)
Supplement: Supplementary file 2 — Informed consent materials. (DOCX 105 kb) [file 13063_2019_3246_MOESM2_ESM.docx]

# Appendices

Informed Consent Materials


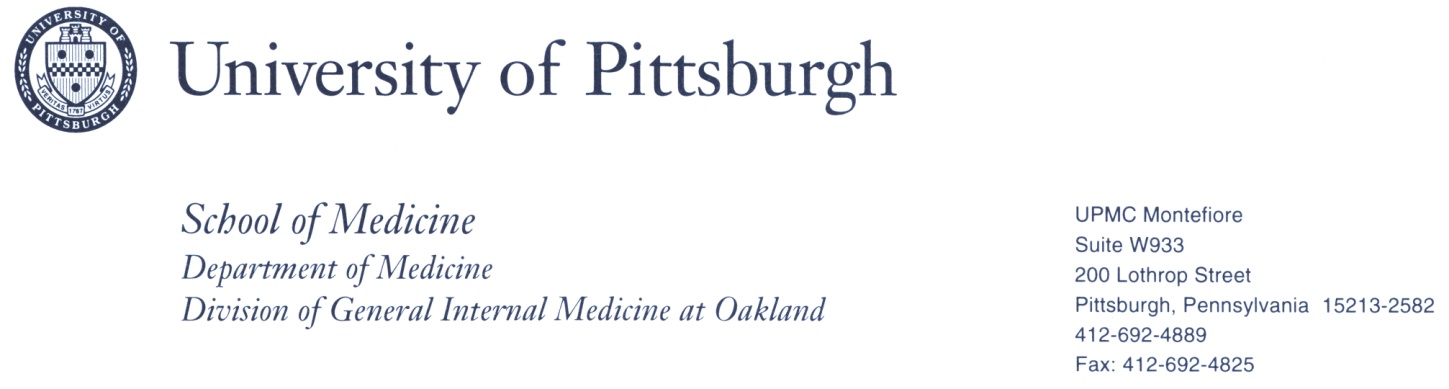
The University of Pittsburgh is doing a research study called Just Kwit. It is testing a smartphone app to see if it can help smokers stay smoke-free after they leave the hospital. This study is funded by the Agency for Healthcare Research and Quality (AHRQ).

We are giving you information about this study because you may be eligible to join it. If you choose to join the study, you have a 50% chance of getting Program A and a 50% chance of getting Program B.

**What will I be asked to do**

Program A:

- We will review your cessation plans after discharge that you have already discussed with your tobacco counselor. If you consented to be contacted by the state’s tobacco quitline, we will send your contact information accordingly.

Program B:

- We will review your cessation plans after discharge that you have already discussed with your tobacco counselor. If you consented to be contacted by the state’s tobacco quitline, we will send your contact information accordingly.
- We will show you a smartphone app that was designed to help smokers quit. We will help you install it on your smartphone and show you how to use its features.

Participants in both programs will be asked to answer a survey at the beginning of the study. You will also be contacted by staff 1 month after you leave the hospital to conduct a second survey about smoking. After you leave the hospital, the survey will be conducted by telephone if you are not able to return for an interview. Data from the smartphone app will also be collected from participants in Program B.

**Compensation**

We will pay you $25 for each survey completed for a total of $50. We will ask you to provide your social security number so that we can pay you for your participation. You may choose to not give us your social security number, but we will deduct 28% of your compensation for tax purposes.

**Potential Risks**

The potential risk to you from participating in the study is minimal. For example, answering some of the survey questions could make you feel uncomfortable, but you have the right to stop participation at any time. Another potential risk would be the inadvertent release of information to someone out of the research study. Although every reasonable effort has been taken, confidentiality during Internet communication activities cannot be guaranteed and it is possible that additional information beyond that collected for research purposes may be captured and used by others not associated with this study. We will take all available precautions to maintain confidentiality and to minimize this risk.

**Privacy and Confidentiality**

Any information about you obtained from this research will be kept confidential (private). All computer-based files will only be made available to personnel involved in the study through the use of access privileges and passwords. The collection of any personal information will be minimal and limited to the amount necessary to achieve the aims of the research. Your identity will not be revealed in any description or publications of this research. In unusual cases, research records may be released in response to an order from a court of law. It is also possible that authorized representatives from the University of Pittsburgh Research Conduct and Compliance Office may review data for the purpose of monitoring the conduct of this study. While we have no current plans to share data, there are potentially other researchers that might join this study to collaborate on manuscripts or grant proposals. Any future collaborators who could access the data will be submitted for IRB approval. .Also, if the investigators learn that you or someone with whom you are involved is in serious danger or potential harm, they will need to inform the appropriate agencies, as required by Pennsylvania law.

**Withdrawal from study participation**

Your participation in this study is voluntary and you can stop at any time. If you decide not to participate in the study, it will not affect medical care you receive now or in the future. If you decide to withdraw from the study, you may contact the research team at 412-692-2578.

A description of this clinical trial will be available on http://www.clinicaltrials.gov, as required by US Law. This website will not include information that can identify you. At most, the website will include a summary of the results. You can search this website at any time.

**Contact information**

Dr. Kar-Hai Chu is the person in charge of this research study. You can call him at 412-692-2578 Monday through Friday, from 8 am to 430 pm. If you have any questions about your rights as a research subject or wish to talk to someone other than the research team, please call the University of Pittsburgh Human Subjects Protection Advocate toll-free at 866-212-2668.

********** Voluntary Consent **********

The above information has been explained to me and all of my current questions have been answered. I understand that I am encouraged to ask questions about any aspect of this research study during the course of this study, and that such future questions will be answered by a qualified individual or by the investigator(s) listed in this consent document at the telephone number(s) given. I understand that I may always request that my questions, concerns or complaints be addressed by a listed investigator.

I understand that I may contact the Human Subjects Protection Advocate of the IRB Office, University of Pittsburgh (1-866-212-2668) to discuss problems, concerns, and questions; obtain information; offer input; or discuss situations that have occurred during my participation. By signing this form, I agree to participate in this research study. A copy of this consent form will be given to me.

_____________________________________ ____________________

Name Date

_____________________________________

Signature

INVESTIGATOR CERTIFICATION:

I certify that I have explained the nature and purpose of this research study to the above-named individual(s), and I have discussed the potential benefits and possible risks of study participation. Any questions the individual(s) have about this study have been answered, and we will always be available to address future questions, concerns or complaints as they arise. I further certify that no research component of this protocol was begun until after this consent form was signed.

___________________________________ ________________________

Printed Name of Person Obtaining Consent Role in Research Study

_________________________________ ____________

Signature of Person Obtaining Consent Date
